# Treatment of pseudobulbar affect (PBA) in a patient with a history of traumatic brain injury, partial brain resection, and brainstem stroke: a case report

**DOI:** 10.1186/s13256-020-02525-3

**Published:** 2020-12-04

**Authors:** Fletcher Graham Young, Diep Nguyen

**Affiliations:** University of Pikeville, Kentucky College of Osteopathic Medicine, The Medical Center, Bowling Green, KY 42101 USA

**Keywords:** Pseudobulbar affect, Stroke, Emotional incontinence, Dextromethorphan/quinidine, Case report

## Abstract

**Background:**

Pseudobulbar affect is a very distressing and underdiagnosed neuropsychiatric disorder that causes contextually inappropriate episodes of laughing and crying and general emotional incontinence. Although many proposed etiologies exist, the most widely accepted theory espouses the disruption of a corticopontine–cerebellar circuit that governs the modulation of emotional response. Pseudobulbar affect is commonly diagnosed secondary to primary neurological disorders such as amyotrophic lateral sclerosis, multiple sclerosis, and traumatic brain injury. Traditional pharmacological treatment of pseudobulbar affect is largely comprised of antidepressant therapy, including tricyclic antidepressants such as amitriptyline and selective serotonin reuptake inhibitors such as fluvoxamine. However, neither of these medication classes has been studied for the treatment of pseudobulbar affect in controlled trials, and their utility remains questionable.

**Case presentation:**

We describe a case of a 62-year-old Caucasian man with history of traumatic brain injury, ischemic brainstem stroke, and depression who developed intractable pseudobulbar affect. This patient’s intensely distressing symptoms were not alleviated by amitriptyline. However, after being placed on fixed-dose 20 mg/10 mg dextromethorphan/quinidine (Nuedexta), our patient experienced complete resolution of his symptoms. He has experienced no deleterious side effects.

**Conclusions:**

This case provides anecdotal evidence for the efficacy of dextromethorphan/quinidine in the treatment of pseudobulbar affect with remarkably swift and complete cessation of symptoms. As a secondary point, it is worth noting that our patient had experienced two devastating neurological traumas, both in anatomical areas that have been implicated in the corticopontine–cerebellar circuit thought to be responsible for pseudobulbar affect. However, only the second trauma, an acute left pontine infarction, produced symptoms of emotional disinhibition. The authors hope that reporting this case will provide both context for physicians managing this condition and hope for patients with this socially and psychiatrically damaging disease.

**Supplementary Information:**

The online version contains supplementary material available at https://doi.org/10.1186/s13256-020-02525-3.

## Introduction

**Fig. 1:**
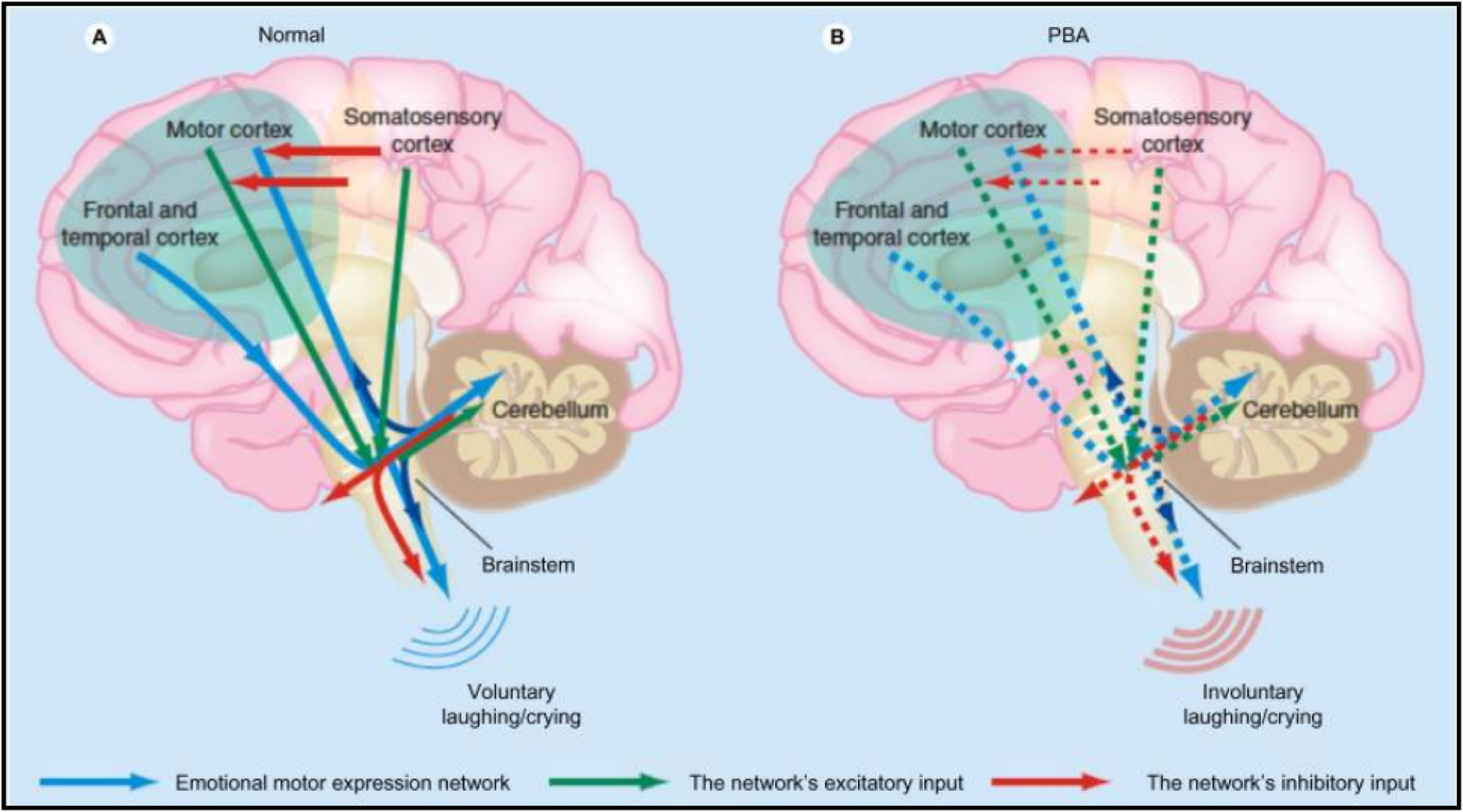
Proposed pathophysiology of pseudobulbar affect. **a** Input from the motor, frontal, and temporal cortices to the brainstem is modulated by input from the cerebellum. Inhibitory input from the somatosensory cortex modulates the motor input. **b** Reduced inhibitory input (*broken red arrows* in the cortex) results in disinhibition, giving rise to inappropriate emotional reactions to stimuli. Permission was granted by Miller et al. (©Taylor & Francis [2]) to reuse this figure

Pseudobulbar affect (PBA) is a neuropsychiatric condition defined by pronounced emotional lability and hypersensitivity to emotional or social stimuli. PBA most often presents as involuntary and inappropriate laughing or crying and occurs out of proportion or incongruent to the patient’s subjective emotional state. Although many proposed etiologies exist, PBA is thought to relate to the release of brainstem emotional control centers from regulation by the frontal lobes [1]. Specifically, the most widely accepted theory maintains that the classic signs of emotional dysmetria are caused by the disruption of a distinct corticopontine–cerebellar circuit that governs the modulation of emotional response [2] (Fig. 1).

PBA is nearly always produced secondary to neurological trauma such as stroke, brain tumor, or progressive neurodegenerative disease such as amyotrophic lateral sclerosis (ALS) [1]. However, because PBA often involves laughing and crying, the condition is frequently mistaken for a symptom of clinical depression, post-traumatic stress disorder, bipolar disorder, or another purely psychiatric condition. Perhaps because of this symptomatic overlap with other common neuropsychiatric conditions, a set of definitive criteria for the diagnosis of PBA has yet to be agreed on. Currently, interested physicians may rely on the published and widely circulated diagnostic criteria of Poeck or Cummings [3, 4], or they may rely on several standardized grading tools that physicians may use to measure the severity of a patient’s symptoms, such as the Center for Neurologic Study-Lability Scale, which is a validated, self-administered questionnaire focused on patient control of laughing and crying [5].

The prevalence of PBA in the United States is thought to be greatly underrecognized [6]. A survey of 8876 patients with commonly associated underlying neurological conditions of Alzheimer disease, ALS, multiple sclerosis (MS), Parkinson disease, stroke, and traumatic brain injury revealed a prevalence of roughly 10% in this population, and estimates of the overall prevalence in the United States are between 1.8 million and 7.1 million, depending on the diagnostic criteria applied [6]. Moreover, it has been estimated that less than half of patients with PBA have received an appropriate diagnosis for this condition [6].

Fixed-dose 20 mg/10 mg dextromethorphan/quinidine ([DM/Q] Nuedexta; Avanir Pharmaceuticals, Aliso Viejo, CA, USA) is the first and only drug approved for the treatment of PBA. Dextromethorphan is the most well known as a noncompetitive *N*-methyl-d-aspartate receptor antagonist, but it has a multifaceted pharmacology and has interactions with serotonin transporters, noradrenaline transporters, sigma-1 receptors, and α_3_β_4_ nicotinic acetylcholine receptors. However, its specific mechanism of action in the treatment of PBA is currently unknown [7]. Quinidine is a class Ia sodium channel–blocking antiarrhythmic, but it is combined with dextromethorphan because of its secondary ability to competitively inhibit cytochrome P450 2D6, which increases and prolongs the plasma levels of dextromethorphan, increasing bioavailability [7]. The most common adverse reactions to DM/Q are diarrhea, dizziness, and cough [8]. It is recommended that patients be monitored for hypokalemia and hypomagnesemia before and during treatment with DM/Q, particularly those with risk factors for QT prolongation. The combination is a pregnancy category C drug and is also contraindicated in patients with a history of atrioventricular block, thrombocytopenia, hepatitis, bone marrow depression, or lupus-like syndrome [8]. Additionally, although DM/Q is currently approved only to treat PBA, it may soon become a far more commonly prescribed medication. Conditions currently being investigated with dextromethorphan as a stand-alone or add-on treatment in clinical trials include Rett syndrome, rheumatoid arthritis, Gulf War illness, diabetic macular edema, attention-deficit/hyperactivity disorder, agitation in depression, schizophrenia, Alzheimer disease, and episodic migraine [9]. In this case report, we describe the utility and efficacy of DM/Q in a patient with severe PBA and significant neuropsychiatric comorbidities, and we advocate for its application in similar clinical scenarios.

## Case presentation

We present a case of a 62-year-old Caucasian man with several neurovascular and psychiatric conditions. The patient was first injured in 1980 in a construction worksite accident, wherein he was struck by a falling cinder block and experienced significant cranial trauma that required a right frontotemporal craniotomy and partial brain resection. The resected bone was then replaced with artificial titanium plating. Following this surgery, the patient remained comatose for a period of 6 days, finally awakening on Christmas Day 1980. Follow-up magnetic resonance imaging and computed tomography confirmed the presence of a large, right-sided cystic encephalomalacia with subsequent gliosis subjacent to the resected brain tissue. Following this initial injury, the patient surprisingly made a full recovery, with no permanent neurological deficits. However, he began to experience painful and intractable headaches. Despite the best efforts of his physicians, these headaches soon became debilitating and made it difficult for the patient to hold a steady job. For the next 30 years, he struggled with his injury, eventually becoming homeless. During one visit to the hospital, he was diagnosed with major depressive disorder and subsequently was started on amitriptyline, which the patient has taken ever since. For most of this period, however, the patient had little or no regular healthcare, and his health slowly deteriorated.

In 2013, the patient, a lifetime smoker, experienced a thromboembolic left pontine infarction. He again recovered, and, interestingly, this event caused a dramatic reduction in the frequency and intensity of the patient’s headaches. However, this time, the patient retained a unilateral right-sided oculomotor nerve palsy and significant speech deficits for years afterward. Several days after this stroke, the patient began to experience episodes of inappropriate laughing and crying and general emotional incontinence. At this time, he was diagnosed with PBA. In addition to his amitriptyline, initial attempts at treatment with several commonly prescribed antidepressants had no effect on the patient’s PBA symptoms. The patient stated that he was not so much bothered by his emotional incontinence in and of itself, but rather by the perception of others in social or everyday public settings. The patient gradually became more socially reserved. Apprehensive of inappropriate and involuntary displays of emotion, the patient started to avoid public settings altogether, such as church and the grocery store. In early 2017, after years of unsuccessful pharmacotherapy, the patient was placed on DM/Q 30 mg by mouth twice daily. At this time, the patient’s vital signs were recorded as follows: blood pressure 116/82 mmHg, weight 296 lb., body mass index 42.47 kg/m^2^, heart rate 82 beats/minute, respiratory rate 20 breaths/minute, body temperature 97.7 °F, and oxygen saturation 93%. Within 1 week, he experienced total resolution of his symptoms. The patient has experienced no negative side effects. Additionally, he reports that if he forgets or is unable to take this medication for 2–3 days, he begins to experience breakthrough symptoms, which quickly resolve after resuming medication.

This patient’s quality of life is now much improved. The authors are happy to report that he is recently married and living comfortably, thanks to federal government benefits. Through great personal effort, the patient has recovered near-full speech ability and is no longer apprehensive of social settings.

## Discussion

Although the precise mechanisms of DMQ in ameliorating PBA are not known, modulation of excessive glutamatergic transmission within corticopontine–cerebellar circuits may contribute to its benefits [2]. Before the approval of DM/Q in 2011, PBA and similar syndromes involving emotional incontinence were, and still are, treated off-label with antidepressants, including tricyclic antidepressants such as amitriptyline and selective serotonin reuptake inhibitors such as fluvoxamine [2]. However, experimental evidence in the form of clinical trials supporting the efficacy of these medication classes in treating PBA is lacking [10]. DM/Q, however, has been proved to be very effective. In a randomized, double-blind, controlled 4-week study of 140 patients with PBA and ALS, DM/Q 30/30 mg was proved to be superior to its individual component drugs [11]. A 12-week, double-blind, placebo-controlled study of DM/Q 30/30 mg showed similar efficacy in 150 patients with MS and PBA. A subsequent 12-week study of patients with PBA and ALS or MS showed superiority to placebo for the 20/10- and 30/10-mg doses [12]. In a 12-week randomized, double-blind trial titled STAR, 326 patients with ALS and MS who had clinically significant PBA were treated twice daily with either DM/Q or placebo [13]. Efficacy was maintained over the length of the trial, an open-label extension (30/10 mg dose), with further improvement of mean efficacy scores [13]. In a 12-week noncomparative cohort study of 120 patients, PRISM II, DM/Q 20/10 mg twice daily also improved PBA secondary to traumatic brain injury [14]. Across these studies, DM/Q was generally safe and well tolerated, with no evidence of clinically relevant cardiac or respiratory effects. Adverse side effects have been described as mostly mild or moderate and include nausea, dizziness, headache, weakness, and gastrointestinal complaints [11, 13].

The success in our patient’s case speaks to the efficacy of DM/Q for the treatment of PBA in acutely affected patients, but it also raises questions regarding the pathophysiological “gate control” model of PBA, which posits the cerebellum as the apparatus responsible for unconscious modulation of emotional expression, scaling it appropriately and producing an emotionally congruent response according to the contextual information transmitted via descending pathways from the sensory cortex through the frontal and temporal cortices [15].Our patient had experienced two devastating neurological traumas, both in anatomical areas that have been implicated in the corticopontine–cerebellar circuit thought to be responsible for PBA. The patient’s first injury, which resulted in the removal of a significant portion of the frontotemporal cortex, should theoretically have interrupted the flow of contextual information to the pons and cerebellum and produced symptoms of emotional disinhibition. However, only the second trauma many years later, an acute left pontine infarction, induced the predicted symptoms of PBA.

With these data in mind and the success in our patient’s case, the authors encourage managing physicians to consider DM/Q for the treatment of PBA in affected neuropsychiatric patients and to keep in mind the clinical variability of this condition.

## Conclusions

Although DM/Q has been proved to be both safe and clinically efficacious in controlled studies, it is a relatively new entrant in the field of neuropsychiatric illness. Although the precise therapeutic mechanism is still a subject of study, it is possible that DM/Q will play a much larger role in the treatment of a diverse array of neurological illnesses in the near future. Its network of neurochemical mechanisms is a subject of ongoing research and has been promoted as a possible candidate for medical therapies for depression, pain, seizures, and methotrexate toxicity [9].

This case provides anecdotal evidence for the efficacy of DM/Q in the treatment of PBA, with a remarkably swift and complete cessation of symptoms in an acutely affected patient. It is the only drug approved for the treatment of PBA, but many physicians are understandably wary of straying from established avenues of pharmacotherapy. The authors hope that reporting this case will provide both context for physicians managing this condition and hope for patients with this socially and psychiatrically damaging disease. In addition, this case underlines the need for a more precise understanding of the pathophysiology of PBA, because the current “gate control” model cannot adequately explain the discrepancy in resultant symptoms between the patient’s two neurological traumas. Further research is required in order to provide a more complete pathological picture of this complex neurological disease.

## Supplementary Information

**Additional file 1:** **Figure 2** Structural formula of dextromethorphan. **Figure 3** Structural formula of quinidine sulfate. **Table 1** Concurrent Medications. **Table 2** History.

## Data Availability

Data sharing is not applicable to this article. No datasets were generated or analyzed for this report.
