# Supplementary material for: Treatment of pseudobulbar affect (PBA) in a patient with a history of traumatic brain injury, partial brain resection, and brainstem stroke: a case report
Source: J Med Case Rep. 2020 Dec 4;14:235. doi: 10.1186/s13256-020-02525-3 (PMC7716484; doi:10.1186/s13256-020-02525-3)
Supplement: Supplementary file 1 — Additional file 1: Figure 2 Structural formula of dextromethorphan. Figure 3 Structural formula of quinidine sulfate. Table 1 Concurrent Medications. Table 2 History. [file 13256_2020_2525_MOESM1_ESM.docx]

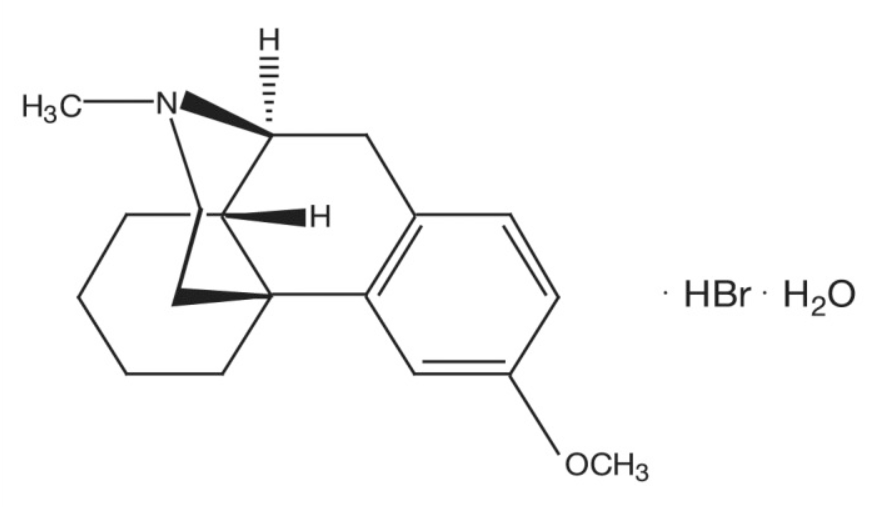
Supplementary Information

**Figure 2** – Structural formula of dextromethorphan^15^


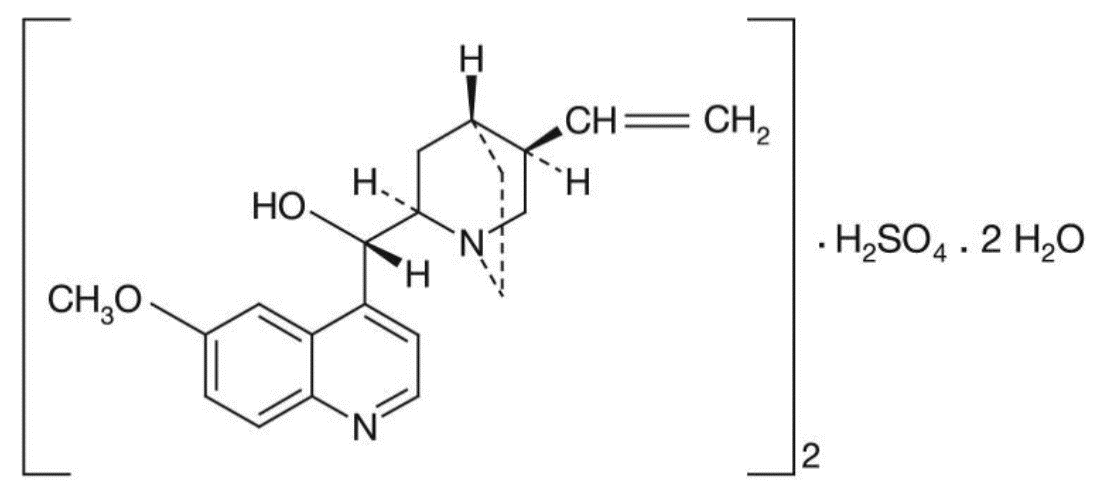


**Figure 3** – Structural formula of quinidine sulfate^16^

| **Past Medical History** | **Past Surgical History** |
| --- | --- |
| Pseudobulbar affect (PBA) | Craniotomy & partial brain resection |
| Essential hypertension | Appendectomy |
| Hyperlipidemia | Tracheostomy |
| Cerebrovascular disease | Cholecystectomy |
| Obesity (BMI 40 - 44.9) |  |
| Major Depressive Disorder |  |
| Hypothyroidism |  |
| Gout |  |

| Nuedexta 30mg PO BID | Coreg 12.5mg PO BID |
| --- | --- |
| Amitriptyline hydrochloride 100mg PO QHS | HCTZ-Lisinopril 25mg-20mg PO QD |
| Amlodipine besylate 10mg PO QD | Levothyroxine sodium 0.112mg PO QD |
| Atorvastatin calcium 40mg PO QHS |  |

*Patient information at start of DM/Q pharmacotherapy*
**Table 1** – Concurrent Medications

**Table 2** – History

**^16^** National Center for Biotechnology Information. PubChem Database. Quinidine sulphate, CID=441326, https://pubchem.ncbi.nlm.nih.gov/compound/Quinidine-sulphate (accessed on May 27, 2020)

**^17^** Haiman, Guy, Hillel Pratt, and Ariel Miller. "Brain responses to verbal stimuli among multiple sclerosis patients with pseudobulbar affect." Journal of the neurological sciences 271.1-2 (2008): 137-147.
